# Ten-year survival in early-stage breast cancer patients in a comprehensive breast cancer care program in India

**DOI:** 10.3389/fpubh.2025.1629401

**Published:** 2025-08-19

**Authors:** Priyansh Nathani, Parth Tailor, Prashant Bhandarkar, Priti Patil, Pratima Pimpalkar, Niranjna Swaminathan, Riya Sawhney, Nobhojit Roy, Anita Gadgil

**Affiliations:** ^1^World Health Organization Collaborating Centre for Emergency, Critical and Operative Care, Program for Global Surgery & Trauma, The George Institute for Global Health, New Delhi, India; ^2^Systems for Trauma and Blood Lab, Program in Global Surgery and Social Change, Department of Global Health and Social Medicine, Harvard Medical School, Boston, MA, United States; ^3^Independent Research and Analytics Consultant, Vadodara, India; ^4^Department of Statistics, Bhabha Atomic Research Centre (BARC) Hospital, Mumbai, India; ^5^Department of Surgical Oncology, Inlaks and Budhrani Hospital, Sadhu Vaswani Mission's Medical Complex, Pune, India; ^6^School of Medicine, Taylor's University Lakeside Campus, Subang Jaya, Malaysia; ^7^Faculty of Medicine and Health Sciences, McGill University, Montreal, QC, Canada; ^8^Department of Global Public Health, Karolinska Institute, Stockholm, Sweden; ^9^Cachar Cancer Hospital and Research Center, Silchar, Assam, India

**Keywords:** breast cancer, survival, early detection, universal health coverage, India

## Abstract

**Introduction:**

Breast cancer accounted for 21.9% of all cancer deaths among women in India in 2020. Fifty seven percent of the breast cancers in India are detected at advanced stages. The lack of adequate resources for diagnosis and treatment adds to the delay and reduces survival. The clinical stage at diagnosis is the most important prognostic factor. Increased cancer awareness, early diagnosis, and affordable and accessible treatment facilities have been recommended for clinical downstaging and improved survival in low- and middle-income countries including India. We implemented a comprehensive breast care program based on these recommendations. This study explores the long-term survival outcomes of patients diagnosed with early-stage breast cancer (EBC) in an early detection program within a universal health coverage (UHC) scheme.

**Methods:**

This is a cohort study of women diagnosed with early-stage breast cancer under the UHC scheme between 2008 and 2018. The follow-up was done through electronic medical records, in-person clinic visits, and telephone calls. The primary outcomes were 5- and 10-year overall survival and disease-free survival.

**Results:**

A total of 185 patients who presented with EBC were recruited among 254 incident breast cancer cases throughout the study period (72.8%). The average overall survival was 123 months. Five-year overall and disease-free survival were 85.2 and 84.6%, respectively. Ten-year overall and disease-free survival were 79.0 and 76.2%, respectively.

**Discussion:**

This study underscores the importance of early detection in breast cancer. It also demonstrates that 5- and 10-year survival rates better than those reported in Indian cancer registries are achievable through comprehensive cancer care and UHC.

## Introduction

Breast cancer is the most prevalent type of cancer among women worldwide, accounting for 11.7% of all cancer cases in 2020. Of these, there were 2.3 million newly diagnosed cases, an increase from 1.67 million in 2012 (1, 2). It is also the leading cause of cancer-related deaths among women. Despite a lower incidence rate of breast cancer among women in India than among those in high-income countries (HICs), the mortality rate in India is similar to the global mortality rate of 13.3 per 100,000 (3). The observed rates of disproportionate mortality can be attributed to the diagnosis of disease at advanced stages and the lack of appropriate early detection, diagnostic, and treatment facilities (4).

The disease stage at the time of breast cancer diagnosis remains one of the most important prognostic factors of survival (5). In India, 57% of cases are diagnosed in the late stage of the disease, leading to a poor 5-year survival rate of up to 52% (6, 7). In HICs, 80%−85% of breast cancer patients are diagnosed with stage I or II disease (8). Early-stage breast cancer (EBC) is associated with a relatively good prognosis with early intervention. As a result, in developing countries such as India, there are marked differences in survival by stage at diagnosis (9).

With a low proportion of cancers being detected at an early stage, there are limited studies from India on EBCs and their long-term survival and follow-up. Additionally, in Indian settings, the post treatment follow-up of patients is a challenge due to a lack of facilitating systems; hence, patients are frequently lost to follow-up. Early diagnosis and affordable and accessible treatment have been recommended for improved survival of patients with breast cancer in India (10, 11). We established a program for the early diagnosis, comprehensive treatment, and follow-up of breast cancer in a population covered by universal health coverage (UHC) in Mumbai, India (12, 13). This manuscript describes the long-term overall survival (OS) and disease-free survival (DFS) of patients diagnosed with EBCs through this program (14).

## Materials and methods

This is a cohort study conducted at an urban community healthcare facility in India, catering to 1,00,000 people. The Government of India's Department of Atomic Energy provides lifelong UHC to its employees and their family members residing in and around Mumbai, India. This healthcare system is implemented through a network of 14 primary healthcare centers (PHCs) across Mumbai and a secondary hospital. We have implemented a comprehensive breast care program for women between the ages of 30 and 69 years under this scheme (13). Comprehensive breast clinics were set up at PHCs and the hospital. Cancer awareness programs and clinical breast examinations (CBE) for self-referred women were implemented through medical officers at these centers. All women with positive CBE findings at PHCs were referred to a community referral hospital, with support provided by PHC staff to ensure high retention across the care continuum. We aimed to increase breast cancer awareness and early detection activities, improve compliance with treatment and ensure consistent follow-up of these patients. An attempt was made to complete diagnostic imaging and cytology on the same day the patient arrived at the breast clinic in the hospital. Treatment was started within 2 weeks of the diagnosis, and adjuvant treatment was started within 3 weeks of surgery. Further details of the UHC scheme, early detection program, and the patient cohort have been published previously (13, 15, 16).

We included women who were diagnosed with stage I, II, of breast cancer in this cohort between January 2008 and December 2018 (17) (Supplementary Table 1). We used the eighth edition of the TNM classification by the American Joint Committee on Cancer (18). The treatment of all patients was decided upon by a multidisciplinary team that followed standard treatment protocols at a tertiary cancer center within the city of Mumbai. Modified radical mastectomy (MRM) or breast-conserving surgery (BCS) was performed according to patient eligibility and patient choice of surgery. All patients achieved complete axillary clearance, and none of the patients underwent sentinel lymph node biopsy. Radiotherapy was given when indicated at the same tertiary cancer care center. The costs were covered under the scheme. Chemotherapy, hormone therapy, and targeted therapy with trastuzumab were given to all eligible patients, irrespective of the economic status of the patients through the UHC. Adjuvant chemotherapy included four cycles of methotrexate, cyclophosphamide, and 5-fluorouracil, followed by four cycles of taxane-based therapy. Hormone receptor-positive patients received tamoxifen or letrozole for 5 years, while those with HER2/neu-positive tumors were given trastuzumab for 1 year. All EBC patients undergoing BCS completed radiotherapy. No patients discontinued or defaulted on treatment. We followed up with the survivors as they visited for routine follow-up appointments in breast clinics conducted at the hospital from 30th Jan 2008 until 30th November 2020. Patients were followed up according to pre-defined institutional protocol—every 3 months during the first 3 years, every 6 months for the subsequent 2 years, and annually thereafter. Patients who missed follow-up visits were identified from the electronic medical records, contacted via telephone, and requested to attend follow-up at the hospital. At these appointments, routine evaluations and detailed clinical examinations were performed by trained medical doctors. Necessary investigations and treatment were provided during these visits. Recurrent cancer was treated as per standard treatment guidelines by multidisciplinary teams at the tertiary cancer center. Comprehensive breast care clinics also provided palliative care on outpatient and inpatient bases as and when indicated. The costs for primary, adjuvant, follow-up, and palliative treatment were covered under the UHC scheme, with no out-of-pocket expenses for patients or their families. UHC and comprehensive breast cancer care across the continuum were central to this study.

The date of death was mapped from telephone discussions with the family or from available electronic medical records for those who died during the follow-up period. The primary outcomes were OS and DFS in the cohort of EBC patients. Other variables included demographic, tumor, and treatment-related factors such as age at diagnosis, menstrual status, education, family history of cancer, tumor size in cm, axillary node status, stage at presentation, histopathological characteristics of the tumor, and adjuvant treatment.

The study was reviewed and approved by the institutional ethics committee before the beginning of the recruitment process (Ethics Committee Number BHMEC/DNB/08/2016).

### Data analysis

We used the Kaplan-Meier (KM) survival analysis method to analyse OS and DFS time-to-event data. OS was considered the length of time from the date of diagnosis to the date of the last follow-up. For those with a distal or local recurrence, the date of recurrence was recorded, and DFS was calculated separately. DFS was considered the length of time from the date of surgery until the date of the last follow-up without recurrence. We also used the KM survival curves to graphically represent the time-to-event probabilities for DFS and OS. Cox proportional hazards regression analysis was conducted to determine the hazard ratios and corresponding *p*-values for each variable for OS and DFS. We used age, education level, menopausal status, family history of cancer, type of surgery, chemotherapy, radiotherapy, hormone therapy, presence of malignant deposits in axillary lymph nodes, and tumor size variables to identify factors associated with OS and DFS among individuals with the specified conditions. All the statistical analyses, graphs, and tabulations were carried out in R.

## Results

A total of 254 patients underwent breast cancer-related treatment under this scheme between 2008 and 2018. Of these, 185 (72.8%) patients were diagnosed with breast cancer at stage I, II, or forming our cohort of EBC patients. There was no drop-out between PHC referral and diagnostic follow-up. The mean age was higher for women who died than for those who survived (66.4 ± 12.5 vs. 60.2 ± 11.7, *p* = 0.005). Ninety (48.6%) of these 185 women underwent BCS, while the remaining 95 (51.4%) were treated with MRM. There were more deaths in the MRM group than in the BCS group (27.3% vs. 11.1%; *p* = 0.004). Twenty-eight (15.1%) deaths occurred during the first 5 years of follow-up. Eight more deaths were observed after the initial period of 5 years, until the end of the study period, totalling 36 (19.5%) cancer-related deaths throughout the study. Table 1 shows the characteristics of the participants.

**Table 1:** Participant characteristics (*N* = 185).

**Variable**	**Category**	**Survived (%)**	**Died (%)**	**Total**	***p*-value**
Total patients		149 (80.5)	36 (19.5)	185	
Age, Mean ± SD		60.2 ± 11.7	66.4 ± 12.5	61.4 ± 12.1	0.005^**^
Average follow-up (months)		73.1 ± 33.63	40.83 ± 29.07	66.82 ± 35.14	
Tumor size	<2 cms	28 (90.3)	3 (9.7)	31	0.321
	2–3 cms	59 (78.7)	16 (21.3)	75	
	3+ cms	62 (78.5)	17 (21.5)	79	
Lymph node status	Negative	98 (80.3)	27 (19.7)	125	0.288
	Positive	51 (81)	9 (19)	60	
Stage of the disease at presentation	Stage 1	27 (93.1)	2 (6.9)	29	0.163
	Stage 2 a	93 (77.5)	27 (22.5)	120	
	Stage 2 b	29 (80.6)	7 (19.4)	36	
Surgery	BCS	80 (88.9)	10 (11.1)	90	0.005^**^
	MRM	69 (72.6)	26 (27.3)	95	
Chemotherapy	Given	56 (78.9)	15 (21.1)	71	0.651
	Not advised	93 (81.6)	21 (18.4)	114	
Radiation therapy	Given	66 (71)	27 (29)	93	0.001^**^
	Not advised	83 (90.2)	9 (9.8)	92	
Hormone therapy	Given	68 (74.7)	23 (25.3)	91	0.049^*^
	Not advised	81 (86.2)	13 (13.8)	94	

^**^p < 0.01; ^*^p < 0.1.

**Figure 1:**
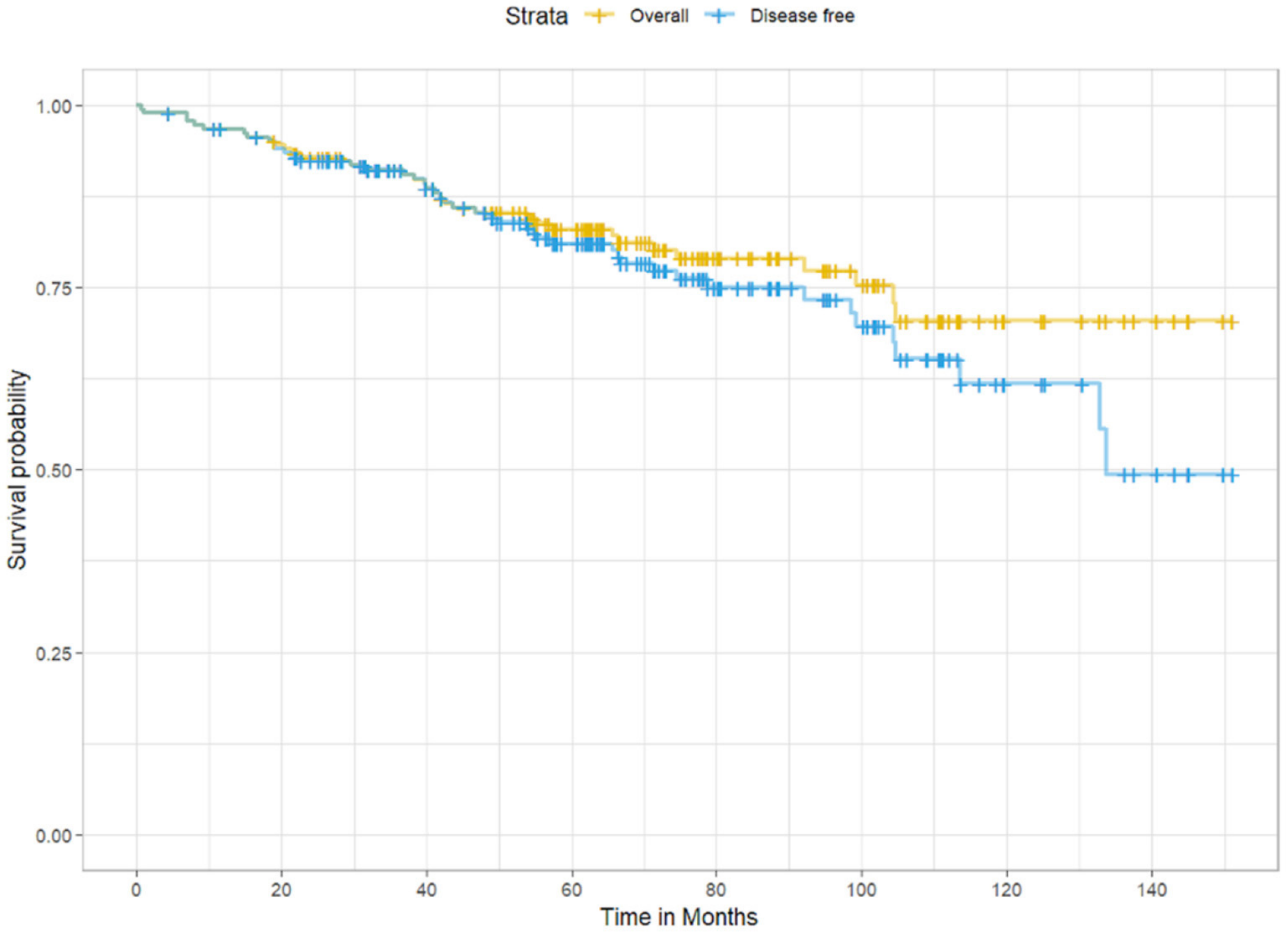
Kaplan-Meier survival curves showing overall survival (yellow) and disease-free survival (blue) among participants in the cohort.

The average OS and DFS were 123 months and 115.2 months, respectively (Figure 1). The OS and DFS rates at 5 years were 85.2 and 84.6%, respectively, whereas at 10 years, they were 79 and 76.2%, respectively.

Table 2 describes the regression analysis of the effect of various sociodemographic factors on survival. Compared with younger women, women in the older age group had a marginally higher risk of death for both OS and DFS.

**Table 2:** Sociodemographic factors affecting overall and disease-free survival.

**Variable**	**Overall survival**		**Disease-free survival**	
	**Crude hazard ratio**	***p*** **-value**	**Crude hazard ratio**	***p*** **-value**
Age	1.043	0.054	1.042	0.0382^*^
Illiterate/education up to school	0.896	0.763	0.826	0.565
Postmenopausal	0.436	0.181	0.477	0.179
Family history of cancer	1.676	0.308	2.489	0.033^*^
Surgery: breast-conserving surgery	0.619	0.286	0.693	0.351
Chemotherapy given	1.242	0.0559	1.452	0.279
Radiotherapy given	0.435	0.068	0.487	0.067
Hormone therapy given	0.53.	0.099	0.469	0.033^*^
Axillary lymph nodes showed malignant deposits	0.756	0.503	0.851	0.662
Tumor size 2–3 cm (ref: TS <2 cm)	2.117	0.240	2.778	0.103
Tumor size 3+ cm (ref: TS <2 cm)	1.847	0.345	2.540	0.144

^*^Statistical significance threshold: *p* < 0.05. TS, Tumor size.

**Figure 2:**
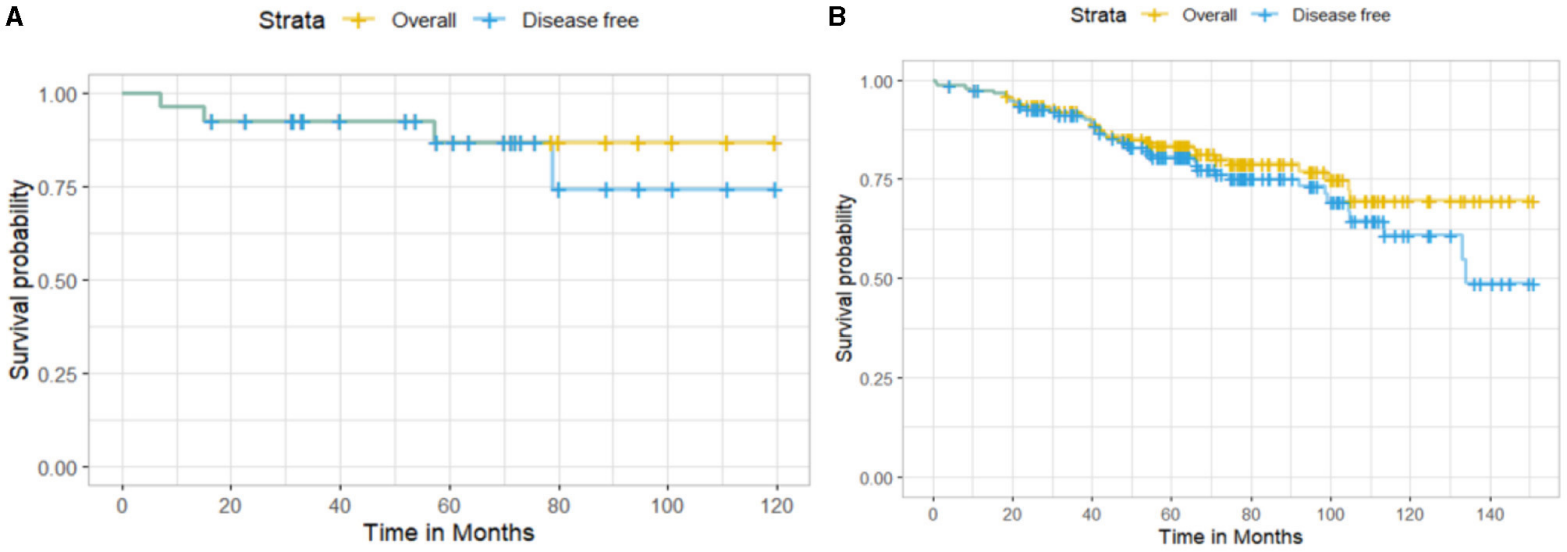
Kaplan–Meier survival curves showing **(A)** overall survival and disease-free survival for HER2neu-positive patients and **(B)** HER2neu-negative patients.

Among the 185 EBC patients, 27 (14.8%) were HER2neu-positive and 156 (84.3%) were negative; two patients had missing values (1.1%). The 10-year OS for HER2neu-positive patients was higher than for HER2neu-negative patients (86.8% vs. 69.8%), with 3 and 31 deaths observed, respectively (Figure 2). However, this difference was not statistically significant (log-rank test: χ^2^ = 0.6, df = 1, *p* = 0.5). Similarly, the 10-year DFS was 74.4% for HER2neu-positive patients (4 recurrences) and 61% for HER2neu-negative patients (40 recurrences), but the difference did not reach statistical significance (log-rank test: χ^2^ = 0.4, df = 1, *p* = 0.5).

**Figure 3:**
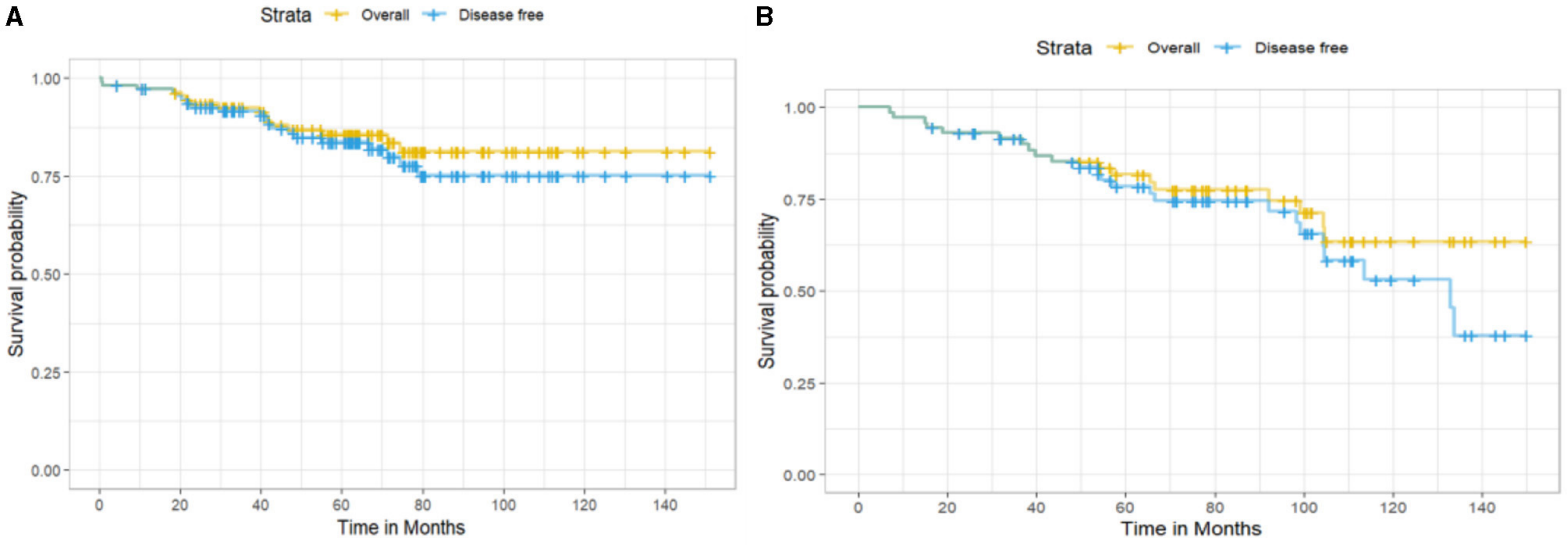
Kaplan–Meier survival curves showing **(A)** overall survival and disease-free survival for hormone receptor–positive patients and **(B)** hormone receptor–negative patients.

Considering hormone receptor status among the 185 EBC patients, 111 (60%) were estrogen or progesterone receptor (ER/PR)-positive, and 72 (38.9%) were negative; two patients had missing value (1.1%). The 10-year OS for ER/PR-positive patients was higher than for ER/PR-negative patients (81.3 vs. 63.4%), with 16 and 18 deaths observed, respectively (Figure 3). However, this difference was not statistically significant (log-rank test: χ^2^ = 1.4, df = 1, *p* = 0.2). The 10-year DFS was 75.2% for ER/PR-positive patients (20 recurrences) and 53.1% for ER/PR-negative patients (24 recurrences), but the difference did not reach statistical significance (log-rank test: χ^2^ = 1.7, df = 1, *p* = 0.2).

## Discussion

A total of 185 (72.8%) of the 254 patients in our cohort underwent surgery in early stages, i.e., stages I, IIa, or IIb. We documented 85.2% OS at 5 years and 79.0% at 10 years in our cohort. The DFS rates were 84.6 and 76.2% at the end of the 5- and 10-year periods, respectively. The average OS and DFS were 123 and 115.2 months, respectively.

Seventy-three percent of cancers in our cohort were diagnosed at early stages. In their randomized controlled trial from India, Mittra et al. (19) reported that 47% of cancers present at advanced stages in their control arm undergoing ‘standard of care' compared to 37% in the intervention arm undergoing CBE. Very few studies in India have documented proportions of EBCs similar to those of HICs, where more than 80% of patients present at early stages (8). A study by Viral et al. (20), in a study from a tertiary hospital in Kerala, India, reported that three-fourths of their recruited patients were diagnosed at early stages. This study was conducted at a tertiary care center in Kerala, which is a state with high literacy rates, high uptake of screening programs, and high overall health indices (21). These factors may have led to higher proportions of health-seeking behavior and EBCs in their cohort, mimicking the HIC scenario. In a comparison study between high- and middle-income countries, Saxena et al. (22) reported a greater percentage of women reporting smaller breast cancers in a HIC (Singapore) than in a middle-income country (Malaysia). There is a scarcity of organized early detection programs in India leading to higher proportions of late-stage cancers at presentation, affecting the OS. A large population-based, randomized controlled trial from Mumbai, India, demonstrated that 63% of breast cancers were detected at stages I and II in the early detection intervention arm compared to 53% in the control arm demonstrating the importance of early detection of breast cancers (19). Our study underlines the importance of organized community-based programs for the early detection of breast cancer.

We reported an OS of 85.2% at 5 years and 79.0% OS at 10 years in our cohort. Our study, based on a structured early detection program covered under UHC, shows the potential to achieve significantly better survival. Data on 10-year OS and DFS, especially for patients with EBCs from India, are scarce. In a systematic review of studies reporting breast cancer survival in India, 16 studies have documented 5-year survival, but none of the included studies had reported 10-year survival (23). Recent findings from the National Cancer Registry Programme (NCRP) in India report a 5-year age-standardized relative survival rate of 66.4% for breast cancer across 11 population-based registries. Ten Year survival was not reported. However, wide regional disparities were noted, with survival rates ranging from 74.9% in the state of Mizoram to as low as 41.9% in the town of Pasighat in Arunachal Pradesh (24). Viral et al. (20) reported a 10-year OS of 79% at 5 years and a 66% OS at 10 years in their tertiary cancer center cohort from Kerala, India, slightly lower than but comparable to our survival data (20). Diagnosing cancer at an early stage, as well as following up with diagnosed cancer patients, are major challenges in India. Resources for early detection and treatment are largely unavailable, and most treatment expenses are out-of-pocket expenditures by the patients. This leads to treatment dropout, loss of follow up and subsequently reduced survival. Hence, 10-year survival data for most LMICs are unavailable, and the reported 5-year survival rates are low, at 40%−50% (25). We followed up with the patients in our cohort through their visits to PHCs. Patients were invited by phone calls for their regular follow-up at the breast clinics. This follow-up of more than 10 years was achieved through a community-level comprehensive early detection and treatment program. Bringing breast cancer detection and follow-up near their homes in the community could play a major role in their health-seeking and long-term survival (16). The reported 5-year survival rates in HICs are >85%, with 80%−85% of cancers presenting at early stages, which is consistent with our findings (8, 26).

In addition to the late stage of presentation, inadequate access to affordable and timely treatment facilities reduces the survival of cancer patients in LMICs. The CONCORD-2 study on cancer surveillance across 67 countries documented large variations in survival between HICs and LMICs and attributed this difference to access to timely diagnosis and treatment facilities (26). Our study was conducted within a population covered by UHC. This approach mitigated the most cited barriers of accessibility and affordability of timely and appropriate care for cancer patients. All the tumors detected by our early detection program at PHCs received priority referrals and free diagnostic and treatment services at the hospital without out-of-pocket expenditures. The high proportion of EBCs in our cohort can be attributed to UHC, sustained awareness drives, systematic CBE at PHCs, and robust referral pathway to tertiary care (16). All recruited women could complete their prescribed treatment, including adjuvant radiation and chemotherapy, without access related barriers or financial toxicity, leading to low attrition rates (25). It has been well documented that secondary and tertiary delays (referring to delays in reaching and receiving care) add to the existing presentation delays in India (27). A study in the north-eastern parts of India by Arvind Kumar et al. (28) reported a treatment delay or tertiary delay of 135 days. They also observed that nearly 80% of patients visited one or two practitioners before reaching the cancer center. Mehrotra et al. (25) observed in their review that system-related delays outweigh presentation delays. Duggan et al. (29), in their study on health system characteristics affecting breast cancer mortality, reported that in 141 out of 148 countries studied, UHC emerged as an important contributor to increased survival in breast cancer patients. Therefore, UHC, which includes comprehensive cancer care, is urgently needed to improve survival. According to 10-year survival data, Viral et al. (20) attributed higher survival rates to the dedicated breast cancer clinics and comprehensive programs.

We reported 85.2% OS and 84.6% DFS at the end of 5 years, suggesting that women survived the recurrence due to continued adjuvant treatment covered under UHC. A retrospective study from a tertiary cancer center in Ghana reported that patients with insurance coverage under which treatment costs were taken care of had an increased survival rate (40%) compared with those without insurance coverage (25%) (30). The breast cancer global initiative has recognized comprehensive cancer management by health system strengthening as the “third pillar” in improving breast cancer survival, with the first two pillars being early detection and timely diagnosis. The initiative advocates for the inclusion of breast cancer care in the national cancer control programs and its inclusion in UHC, especially in LMICs (31). Our study also emphasizes the importance of UHC in cancer care for improving the long-term survival of cancer patients in general and of breast cancer patients.

### Strengths and limitations

This is one of the few studies documenting 10-year survival rates in breast cancer patients in India. The context of implementing comprehensive breast cancer care and equitable access to free healthcare under a UHC scheme, catering to a population of 1,00,000 people is a unique strength of this study. A limitation of our study is that the sample size of our cohort was small compared to that of studies from population-based registries. Additionally, rolling enrolment between 2008 and 2018 led to variation in follow-up durations, and patients with <10-year follow-up were appropriately right-censored in the KM analyses. As a result, the 10-year OS and DFS estimates are based on survival projections rather than complete follow-up for all patients. Moreover, increased community awareness and expanded uptake of the UHC scheme in later years may have contributed to higher recruitment during that period, potentially affecting the representativeness of long-term survival estimates.

### Future directions

This study highlights future implementation priorities to scale up population-based early detection to downstage the disease at presentation. We advocate for the inclusion of diagnostic tests and treatment of breast cancer to enhance access to affordable care and reduce attrition, as more countries are moving toward accepting and delivering UHC to their people. While the UHC model described in this study may not yet be feasible at all centers, India's national health insurance schemes such as the Pradhan Mantri Jan Arogya Yojana (PM-JAY), which includes cancer care, represent promising steps toward expanding access and enabling replication of such programs.

## Conclusion

This study underscores the critical role of early detection and comprehensive cancer care in improving long-term survival outcomes for breast cancer patients. Our observed 5-year OS rate of 85.2% surpasses the national average reported by Indian cancer registries, highlighting the potential benefits of implementing structured early detection programs and ensuring equitable access to treatment under UHC.

## Data Availability

The raw data supporting the conclusions of this article will be made available by the authors, without undue reservation.
