# Supplementary material for: Ten-year survival in early-stage breast cancer patients in a comprehensive breast cancer care program in India
Source: Front Public Health. 2025 Aug 19;13:1629401. doi: 10.3389/fpubh.2025.1629401 (PMC12402064; doi:10.3389/fpubh.2025.1629401)
Supplement: Supplementary file 1 [file Table_1.docx]

**Supplementary Table 1.** Year-wise distribution of early breast cancer EBC patients recruited from 2008 to 2018

| **Year of Diagnosis** | **Number of EBC Patients** |
| --- | --- |
| 2008 | 11 |
| 2009 | 12 |
| 2010 | 9 |
| 2011 | 17 |
| 2012 | 13 |
| 2013 | 19 |
| 2014 | 26 |
| 2015 | 30 |
| 2016 | 18 |
| 2017 | 15 |
| 2018 | 15 |
| Total | 185 |
